# The Unique Role of Peer Support: Exploring the Effects of Various Sources of Social Support on the Mental Health of Unaccompanied Children in China under Residential Education

**DOI:** 10.3390/children10081326

**Published:** 2023-08-01

**Authors:** Lizhang Dong, Yanan Peng, Ran Zhang, Kang Ju, Juzhe Xi

**Affiliations:** 1 Shanghai Key Laboratory of Mental Health and Psychological Crisis, Affiliated Mental Health Center (ECNU), Positive Education China Academy (PECA) of Han-Jing Institute for Studies in Classics, Juzhe Xi’s Master Workroom of Shanghai School Mental Health Service, School of Psychology and Cognitive Science, East China Normal University, Shanghai 200062, China; 51193200073@stu.ecnu.edu.cn (L.D.); 52203200018@stu.ecnu.edu.cn (Y.P.); 10150170222@stu.ecnu.edu.cn (R.Z.); 2 Affiliated Mental Health Center (ECNU), Shanghai Changning Mental Health Center, Shanghai 200335, China; jukang2009@163.com

**Keywords:** unaccompanied children, social support, mental health, resilience

## Abstract

This study investigates the impact of various sources of social support on the mental health of unaccompanied children under residential education in China. Unaccompanied children refer to those whose parents are still alive but unable to raise them due to various reasons. The study utilized self-reported questionnaires administered at two time waves, with the first wave (T1) evaluating family support, teacher support, and peer support, and the second wave (T2) evaluating depression, subjective well-being, and resilience. A total of 202 participants completed both surveys. To examine the predictive effect of different sources of social support on the mental health of these children, the study used the structural equation model with depression and subjective well-being as indicators. The results show that neither family support nor teacher support (T1) had a significant effect on the mental health (T2) of the children. However, peer support (T1) had a significant positive predictive effect on mental health (T2), indicating the unique role of peer support in promoting the mental health of unaccompanied children. The study also explored the mediating role of resilience between social support and well-being, revealing that though the direct effect of teacher support (T1) on mental health (T2) was not significant, the indirect mediating effect of resilience on the relationship between teacher support and mental health was significant. Both the direct and indirect effect of family support (T1) on mental health (T2) were not significant. These findings highlight the importance of creating a positive peer environment for unaccompanied children to promote their mental health. This study has important practical implications for the development of effective intervention programs aimed at improving the mental health of this population.

## 1. Introduction

### 1.1. Unaccompanied Children in China

In China, unaccompanied children refer to a specific population of children whose parents are still alive but unable to care for them due to various reasons. Unlike the Western world, where unaccompanied minors are often immigrants fleeing from war, poverty, or exploitation, or searching for family members [1,2], unaccompanied children in China receive inadequate care due to reasons such as severe disability, serious illness, imprisonment of both parents, or the death of one parent with the other parent facing one or more of the aforementioned challenges [3]. These children are primarily located in mountainous and rural areas in China [4,5,6], where they experience impoverished living conditions and lack necessary social support networks crucial to their development.

As a result of these hardships, the mental health of unaccompanied children in China has been significantly challenged. Both Chinese research on left-behind children [7,8] and international research on children in distress [2,9,10,11,12,13] have demonstrated that unaccompanied children exhibit higher levels of anxiety, depression, and loneliness, as well as lower life satisfaction. Research conducted in China has also revealed that unaccompanied children generally face psychological problems, such as low life satisfaction, depression, feelings of loss, low levels of interpersonal trust, and emotional instability [14,15,16]. Thus, the mental health of unaccompanied children necessitates immediate attention.

### 1.2. Social Support and Children’s Mental Health

Social support plays a vital role in children’s mental health. It encompasses both tangible and emotional assistance that individuals receive through interactions with others in their social networks [17,18]. Such support can make children in distress feel cared for and help them better cope with stress and difficulties, and is widely recognized as one of the most critical external factors affecting children’s mental health [19,20]. Recent research has highlighted the central role of enhancing the social support system in protecting unaccompanied children [4,5,21].

### 1.3. Different Sources of Social Support

Previous research has compared the impact of social support from various systems, such as relatives, family members, teachers, and peers on the mental health of children in distress, such as orphans [20,22,23] and left-behind children [19,24]. Most of the research emphasized the importance of family members, caregivers, and teachers. However, it is challenging to directly apply these findings to unaccompanied children who may have unique needs and challenges due to different social environments and backgrounds. In the meantime, most studies on unaccompanied children have not focused on the relative impact of different sources of social support on the mental health of unaccompanied children in China. To provide unaccompanied children with more accurate and effective support, it is increasingly important to examine different sources of social support and their individual contributions to the mental health of these children.

### 1.4. Peer Support for Unaccompanied Children: Under the Mode of Residential Education

This study examines the role of peer support for unaccompanied children living in two non-profit boarding schools in south-central China. Residential education refers to a type of educational program where students live on campus or in a residential facility while attending school. It has been found to be an effective mode for rural children to establish deep friendships in school [15,25]. Furthermore, compared to non-residential alternatives, residential education is associated with better learning, relationships, and behavioral adaptation [16,24,25]. However, support from teachers in such schools may be uneven [24], and unaccompanied children may have a more isolated cultural and social experience [25].

Thus, this study assumes that peer support is of great and unique value in the social support system of unaccompanied children, especially those in residential education settings.

Firstly, peer support is the most stable and accessible form of social support for children under residential education. According to ecological systems theory [26], the microsystem for these children is primarily the boarding schools they live in. In these schools, peer support and teacher support are the two most important sources of support in schools. However, due to staff shortages, school teachers may find it difficult to care for all students equally. Moreover, in rural areas, high staff mobility can make it difficult to ensure the consistency and stability of teacher support [24]. Therefore, peers who study, live, and grow together provide the most accessible and reliable source of support.

Secondly, peer support meets the needs of school-age unaccompanied children in different development phases. To begin with, Erikson’s eight-stage theory of personality development [27] states that the core task of children’s development between the ages of 6 and 12 is resolving the conflict between diligence and inferiority. Peer support in school can help unaccompanied children in this stage and rebuild their sense of belonging, worth, social confidence, and skills, which can then aid in resolving this conflict. In addition, peer support is crucial for children aged 12 to 18, who are notably building their self-identity. This is especially the case for unaccompanied children, as they may have experienced trauma in their biological families, which can negatively impact their sense of self and lead to problems like self-identity stigmatization. For these children, weaker peer support may lead to lower self-esteem, which is particularly detrimental. However, with adequate peer support, children in residential education can reconstruct their self-identity through establishing healthy relationships with peers who have shared experiences or similar situations [28].

Thirdly, peer support is an important medium for the transmission of values in residential education. The theory of group socialization [29] highlights the significant role of peer groups in cultural transmission during children’s socialization processes. In many cases, the words and actions of peers are more influential than those of teachers and parents. In the case of unaccompanied children in residential education, who may have limited external contacts, their relationships with peers at school become even more important [25], amplifying the influence of peer groups. By paying attention to peer support, educators and practitioners can make better use of its positive influence on the well-being and development of these children.

Finally, peer support is closely related to children’s mental health. On the one hand, a higher level of peer support can directly improve children’s daily mental health [30,31] and mitigate the negative effects of stressful events such as rejection and bullying [22]. On the other hand, the quality of peer relationships can compensate for the absence or low quality of parent–child bonding [23,32,33,34,35]. Thus, peer support not only acts as a mental health booster and stress buffer, but also compensates for a lack of social support from other sources, which is particularly important for unaccompanied children who may face challenges in obtaining sufficient social support.

Based on the reasons mentioned above, we expect that peer support will be a stronger predictor of mental health outcomes in unaccompanied children compared to other forms of support.

### 1.5. The Mediating Role of Resilience

One potential mechanism through which social support enhances mental health for children facing adversity is by fostering resilience. According to the integrated resilience model [36], the interplay between external environmental factors and individual internal resilience factors leads to varying developmental outcomes in individuals. In recent studies, children’s resilience has been considered as a mediating variable of social support’s effect on their mental health [19,23]. Therefore, apart from exploring the relationship between various sources of social support and the mental health of unaccompanied children, this study will also investigate the mediating role of resilience in these relationships. Therefore, two hypotheses were developed in this study:

**H1:**  *Among three sources of social support, peer support would be the strongest predictor of mental health of unaccompanied children under residential education.*

**H2:**  *Unaccompanied children would perceive more social support, which predicted higher levels of resilience that could improve their mental health outcomes.*

## 2. Method

### 2.1. Participants

The data for this study were collected from two public welfare schools located in Hunan province, China. In both schools, Children are mostly enrolled in the third grade. During the semester, they study and stay in school, and maintain contact with their current caregivers through the school’s telephone. During holidays, students return to their homes. A total of 244 children and their caregivers provided informed consent and completed questionnaires in two waves. After quality checking, we finally collected 202 valid questionnaires. The testing materials and procedures were approved by the Human Experiment Ethics Committee of East China Normal University (approval number: HR2-1086–2020, approval date: 12 March 2021). The sample consisted of 108 boys and 94 girls with an average age of 11 years (range: 8–15).

### 2.2. Procedure

This study was conducted with two rounds of data collection performed separately. In both rounds, participants who agreed to participate in the study were concentrated in their classrooms. Researchers included 5 graduate students and a doctoral student in the psychology major, and all of them had been trained in the testing process before participating in the study. After distributing the questionnaires to each participant, for third and fourth grade participants, researchers read each item of the questionnaire twice to make sure children could understand. Participants from other grades read and answered on their own. After participants finished answering, they raised their hands and researchers would collect the questionnaires one by one.

**Figure 1:**
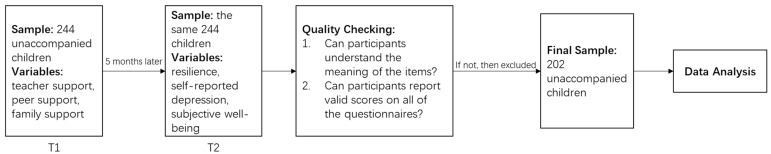
Flowchart of the procedure.

The first round of data collection, referred to as T1, was conducted at the end of the first semester, and the second round, referred to as T2, was conducted 5 months later at the end of the second semester. At T1, data were collected from 244 unaccompanied children in grades 3 to 8 from the two schools on teacher support, peer support, and family support. At T2, data were collected from the same 244 individuals on resilience, self-reported depression, and subjective well-being. After that, we excluded participants who could not understand the meaning of the questions or who failed to effectively report their scores on the questionnaires above. Finally, 202 valid questionnaires were collected (Figure 1).

### 2.3. Measures

Questionnaire on social support status of left-behind children. The scale was compiled by Zhao [24] to assess children’s perceptions of social support from various sources, including government and school support, school administrative support, teacher support, peer support, and family support. For this study, three dimensions were selected: teacher support (7 items), peer support (8 items), and family support (6 items). Our pilot study found that when using the word “parent” in the original scale, many of participants had difficulty answering these questions and did not know how to answer them, as they lack parental care. Thus, the item description of family support was modified according to the actual situation of unaccompanied children (“parent” replaced with “family members”). The questionnaire uses a scale of 1 to 5 points, ranging from “completely disagree” to “completely agree”. The higher the score, the higher the social support children perceive from a particular source. The overall Cronbach’s α coefficient of the scale in this study is 0.889, with the Cronbach’s α coefficient of the family support subscale at 0.746, the α coefficient of the teacher support subscale at 0.844, and the α coefficient of the peer support subscale at 0.877. To test the validity of the scale, confirmatory factor analysis was conducted, and the results are as follows: χ^2^/df = 1.37, RMSEA = 0.043, CFI = 0.952, TLI = 0.946.

“Delighted/Terrible” (D/T) Scale. The subjective well-being of participants is measured using the single-item face scale developed by Andrews and Withey [37]. The scale consists of seven faces, each with a different expression, ranging from very happy to very sad.

Children’s Depression Inventory (CDI). The questionnaire, compiled by Kovacs [38], assesses children’s depression status and is suitable for ages 7 to 17 years old. The reliability and validity of the Chinese version have been tested and confirmed [39]. The scale consists of 27 items, each of which consists of three sentences. The degree of description of each sentence is different, including general response, moderate depressive symptoms, and severe depressive symptoms, which correspond to scores of 0, 1, and 2, respectively. A higher score on the scale indicates a higher level of depression in the participant. The Cronbach’s alpha coefficient for this scale in this study was 0.840. To test the validity of the scale, confirmatory factor analysis was conducted, and the results are as follows: χ^2^/df = 1.28, RMSEA = 0.038, CFI = 0.911.

The Connor-Davidson Resilience Scale(CD-RISC-10). The scale, extracted by Campbell-Sills and Stein [40] from 25 items compiled by Connor-Davidson based on the trait theory of resilience and localized by Wang, et al. [41], covers 5 factors: ability, negative emotions endurance, change acceptance, control, and mental influence. It uses a five-point scale, with 0 for “never”, 1 for “rarely”, 2 for “sometimes”, 3 for “often”, and 4 for “almost always”. The higher the total score, the stronger the child’s resilience. The Cronbach’s alpha coefficient for this scale in this study was 0.821. To test the validity of the scale, confirmatory factor analysis was conducted, and the results are as follows: χ^2^/df = 1.86, RMSEA = 0.065, CFI = 0.941.

### 2.4. Data Analysis

For this study, the data were processed and analyzed using SPSS 23.0 and AMOS 23.0 statistical software. The mean and standard deviation of social support, subjective well-being, self-reported depression, and resilience levels of unaccompanied children were first investigated. The effects of demographic variables, such as gender, age, grade, number of siblings, and parental marital status, on the subjective well-being, self-reported depression, and resilience of the children were then analyzed using one-way ANOVA and Pearson correlation analysis. Additionally, the correlation between family, teacher, and peer support at T1 and the subjective well-being, depression level, and resilience level of children at T2 was investigated.

To examine the mediating effect of resilience between social support and children’s mental health, structural equation modeling (SEM) was conducted as a powerful multivariate analytical tool [42]. Family support, teacher support, peer support, and mental health were measured as latent variables. The 6 items of the family support subscale were regressed onto the latent variable of family support, the 7 items of the teacher support subscale were regressed onto the latent variable of teacher support, the 8 items of the peer support subscale were regressed onto the latent variable of peer support, and the total scores of subject well-being and depression level were regressed onto the latent variable of mental health. In this model, family, teacher, and peer support were independent variables, resilience was a mediating variable, and mental health was the dependent variable. The model fit was evaluated by the following indexes: the χ^2^/df value; the root mean square error of approximation (RMSEA); and the comparative fit index (CFI). To confirm a good model fit, the χ^2^/df should be less than 3.0, the CFI value should be larger than 0.90, and the RMSEA values should be smaller than 0.08 [43].

To test the significance of the indirect effects in the mediation model, we conducted bias-corrected bootstrap tests with a 95% confidence interval (CI). The mediation analysis was evaluated using 2000 bootstrap samples to calculate the 95% bias-corrected and accelerated bootstrap CIs, a method that is considered more statistically robust than traditional approaches [44]. A significant effect is identified if the 95% CI does not include 0.

## 3. Results

### 3.1. The Descriptive Statistics of Demographic Variables of Unaccompanied Children in Residential Education

Table 1 presents the information about participants’ gender, grade level, number of siblings, and their parents’ marital status in this study. Overall, our participants are distributed between third grade to eighth grade (23% are middle school students), with an average age of 11 years. According to the information provided by the principal and teachers, the children of both schools were screened according to certain admission criteria, and all meet the criteria for unaccompanied children in China [3]. The parents of these children may be unable to raise their children alone due to death (11.9%), departure of their partners, or divorce (13.9%), and children with both parents (13.4%) are often unable to be well cared for due to poverty, illness, and imprisonment. All the demographic variables had no significant effects on the subjective well-being, depression, and resilience of unaccompanied children.

### 3.2. Subjective Well-Being, Depression Level, and Resilience Level of Unaccompanied Children in Residential Education

Table 2 presents the means and SDs and correlations of unaccompanied children’s teacher support, peer support, and family support at T1 and their subjective well-being, depression levels, and resilience levels at T2.

The results reveal that teacher support at T1 was not significantly associated with children’s subjective well-being at T2 (*p* > 0.05). Furthermore, children’s teacher support was negatively correlated with depression levels at T2 (r = −0.35, n = 202, *p* < 0.001), and was positively correlated with their resilience at T2 (r = 0.39, n = 202, *p* < 0.001), indicating that the mental health and resilience of unaccompanied children under residential education are partially influenced by their teachers’ support.

Family support of unaccompanied children at T1 was positively correlated with children’s subjective well-being (r = 0.15, n = 202, *p* < 0.05) and resilience (r = 0.27, n = 202, *p* < 0.001) at T2, though weakly (r < 0.3). Additionally, family support at T1 was negatively correlated with children’s depression levels at T2 (r = −0.21, n = 202, *p* < 0.001). These results suggest that the mental health and resilience of unaccompanied children under residential education are weakly correlated to their family support.

Furthermore, peer support at T1 was positively correlated with children’s subjective well-being (r = 0.21, n = 202, *p* < 0.001) and resilience (r = 0.37, n = 202, *p* < 0.001) at T2. Additionally, peer support was negatively correlated with children’s depression levels at T2 (r = −0.39, n = 202, *p* < 0.001). These findings indicate that the mental health and resilience levels of unaccompanied children in residential education are positively influenced by their peer support.

### 3.3. Validation of the Measurement Model

The measurement model in this study includes four latent variables: family support, peer support, teacher support, and mental health. The measurement model was tested and found to fit well according to several indicators, χ^2^ (243, n = 202) = 338.64, CFI (0.941) is greater than the critical value of 0.9, and RMSEA (0.044) is less than the critical value of 0.08. The standard factor loading of all the observed variables are ranged from 0.493 to 0.885 (ps < 0.001), which means the observed variables adequately represented their respective latent variables in the model.

### 3.4. Test of Predictive Effect of Peer, Family, and Teacher Support on Mental Health of Unaccompanied Children: The Mediating Role of Resilience

**Figure 2:**
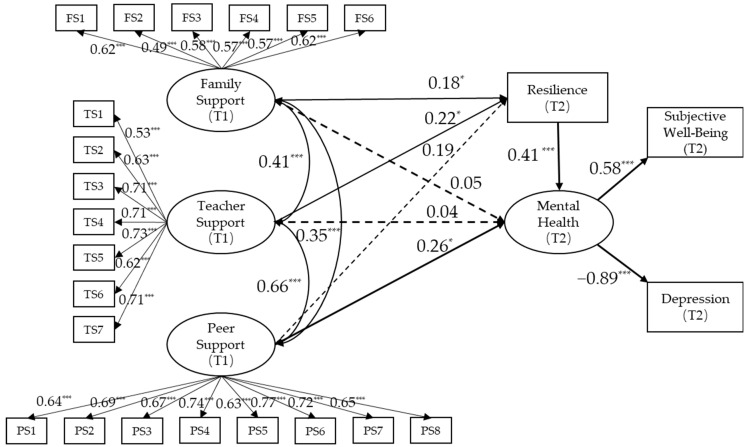
Test of predictive effect of support from three types of sources (family, teachers and peers) on mental health of unaccompanied children: the mediating role of resilience. Note: FS = Family Support, TS = Teacher Support, PS = Peer Support. * *p* < 0.05 (two-tailed); *** *p* < 0.001 (two-tailed).

To test the predictive effect of peer, family, and teacher support on the mental health of unaccompanied children, a model inspection was conducted using social support data at T1 and mental health and resilience data collected at T2 (Figure 2). The results showed that the model fit well with the following indices: χ^2^/df = 1.502, CFI = 0.941, TLI = 0.933, RMSEA = 0.044.

The bias-corrected bootstrap test results regarding the mediation effects are shown in Table 3. There was a significant total effect (β = 0.335, *p* = 0.007) and direct effect (β = 0.258, *p* = 0.047) between peer support and mental health of unaccompanied children under residential education. Moreover, there was a significant indirect effect between teacher support and children’s mental health (β = 0.090, *p* = 0.029). There was neither direct effect nor indirect effect between family support and mental health outcomes.

These findings support the hypothesis of this study, suggesting that peer support, compared to family and teacher support, has the strongest predictive effect on the mental health of unaccompanied children. Additionally, resilience plays a mediating role in the relationship between children’s teacher support and their mental health. 

## 4. Discussion

The present study aimed to investigate the effects of teacher, family, and peer support on the mental health of unaccompanied children under residential education, as well as the mediating role of resilience. The results indicate that among the three types of social support, only peer support at T1 had a significant positive predictive effect on mental health at T2. The findings also suggest that resilience plays a mediating role in the relationship between teacher support and unaccompanied children’s mental health. These results highlight the unique role of peer support in the social support system for unaccompanied children and have implications for evidence-based social policy and social work intervention aimed at promoting the mental health of unaccompanied children in China. Overall, this study contributes to the growing body of literature on the social support system and mental health outcomes of unaccompanied children in China, and sheds light on effective interventions to improve their mental health.

### 4.1. Peer Support Plays a More Important Role than Teacher or Family Support in Predicting the Mental Health of Unaccompanied Children

Consistent with previous research [19,22,45], this study found that social support from family members, teachers, and peers was significantly positively correlated with the subjective well-being and resilience of unaccompanied children, and negatively correlated with their depression level. These results underscore the importance of social support for the mental health of unaccompanied children.

Of the three sources of social support, only peer support was significantly correlated with the subjective well-being, resilience, and depression level of unaccompanied children simultaneously. Specifically, when family, teachers, and peer support were included in the same model, only peer support had a significant predictive effect on the mental health outcomes of unaccompanied children. Therefore, it is reasonable to conclude that, among the three sources of social support that unaccompanied children have direct contact with, peer support is the most important for their mental health.

This conclusion differs from some previous studies examining children in distress, such as orphans [20,22,23], which emphasized the important role of family members, caregivers, and teachers in children’s mental health. However, given the unique situation of unaccompanied children, researchers must take into account their specific needs and experiences. Unlike orphans, unaccompanied children may have complex emotions toward their families and may be unwilling to express their needs to their family members. Additionally, the high turnover of staff in schools makes it difficult for teachers to provide personalized care for all students. Therefore, peers become the most accessible source of social support for unaccompanied children under residential education, and peer support is more unique and important in their social support system.

In the future, interventions to promote the mental health of unaccompanied children could consider the construction of a peer support system in order to provide more effective support. These findings can also inform evidence-based social policy and social work interventions to improve the mental health outcomes of unaccompanied children in China.

### 4.2. Resilience as a Mediator of the Impact of Teacher Support on the Mental Health of Unaccompanied Children under Residential Education

Consistent with previous research on other vulnerable children [19,23,46], this study also found that resilience has a protective effect on the mental health of unaccompanied children and mediates the effect of social support on their mental health. The results show that teacher support can affect children’s mental health by improving their resilience. This indicates that changes in children’s resilience may be a mechanism by which some sources of social support work, supporting the integrated resilience model developed by Kumpfer [36] that emphasizes the interaction between the environment and internal resilience factors in promoting children’s mental health.

Kohut’s theory [47] offered valuable insight into comprehending how teacher support fosters mental health by enhancing resilience. According to Kohut, children have a need to idealize their caregivers, perceiving them as powerful figures. This “idealized route” fosters feelings of comfort and security, thus promoting healthy self-development. In the case of unaccompanied children under residential education, viewing teachers as powerful and identifying with a sense of belonging may instill hope and enable them to seek assistance when necessary, thus making them more resilient in harsh situations, given that they can hardly expect their family members to set the idealized example. 

It is important to note that the study reveals that resilience did not mediate the impact of peer support on children’s mental health, suggesting that the influence of peer support on mental health involves a more complex mechanism.

### 4.3. Research Limitations and Implications

This study has provided insights into the implications of different sources of social support for unaccompanied children, particularly those under residential education. By comparing the effects of different sources of social support on the mental health of these children, we have been able to refine the scope of social support and discuss the mechanism of resilience in this context.

The results of this study have important practical implications for the construction of social support systems for unaccompanied children. In particular, the construction of a peer support system should be given priority. However, the current rescue mode still mainly relies on external resources, such as good school supplies and teachers, while the resources within the school, such as the peer support system, may be overlooked. Based on the present findings, managers and teachers of schools for unaccompanied children should invest more in discovering peer resources within the school. For instance, the current support that involves older children taking care of younger children in the dormitory is a creative attempt. Still, additional group activities in school can be organized to strengthen the peer support systems. These practical initiatives can lead to the improvement of the mental health and resilience of unaccompanied children under residential education.

Several limitations should be taken into account when interpreting the findings of this study. Firstly, the small sample size limits the generalizability of the findings, as the specific environment in which unaccompanied children in China reside makes it difficult to collect larger samples. Future research with larger sample sizes could yield more comprehensive results. Secondly, as the sample size is small, in order to ensure the statistical reliability of the results, this study did not consider the impact of differences between the two schools. However, the two schools are close in distance, have the same educational philosophy and modes, and belong to the same foundation. The difference between the two schools may have little impact on results. Future research can include consideration of the school affiliation’s bias. Thirdly, due to the self-reported method used in this study, younger children (below grade 3) who lack sufficient reading and writing abilities were not included in the sample. Moreover, this study is limited to examining the effect of social support on the mental health of unaccompanied children under residential education. Future research could investigate whether social support from different sources has varying effects on the mental health of unaccompanied children in different environments.

## 5. Conclusions

Social support, including support from family members, teachers, and peers, is correlated with the mental health and resilience of unaccompanied children under residential education. Among them, peer support was found to be the most important form of social support in promoting the mental health of unaccompanied children, as it was the only source of social support that directly predicted mental health of unaccompanied children after 5 months. Future interventions aimed at promoting the mental health of unaccompanied children should focus on building strong peer support systems for these children.

## Figures and Tables

**Table 1:** Descriptive statistics of demographic variables of unaccompanied children (n = 202).

	Frequency (n)	Percentage (%)
Gender		
Male	108	53.5
Female	94	46.5
Age	Mean = 10.92 (S.D. = 1.62)	
Grade		
Third grade	40	19.8
Fourth grade	45	22.3
Fifth grade	51	25.2
Sixth grade	20	9.9
Seventh grade	22	10.9
Eighth grade	24	11.9
Siblings	Mean = 3.09 (n = 90, S.D. = 1.30), Max = 8, Min = 1	
Parents’ marital status		
Single	5	2.4
Married and living together	27	13.4
Married and not living together	13	6.4
Divorced and single	28	13.9
Widowed	24	11.9
Remarried	10	5.0
Unknown	95	47.0

**Table 2:** Means, SDs and correlations among social support at T1 and subjective well-being, depression level, and resilience at T2 (n = 202).

	Mean	S.D.	Teacher Support	Peer Support	Family Support	Subjective Well-Being	Depression Level	Resilience
T1								
Teacher Support	25.90	6.21	1					
Peer Support	25.93	7.55	0.58 **	1				
Family Support	20.47	4.71	0.32 **	0.28 **	1			
T2								
Subjective Well-Being	4.76	1.45	0.14	0.21 **	0.15 *	1		
Depression Level	17.60	7.66	−0.35 **	−0.39 **	−0.21 **	−0.52 **	1	
Resilience	21.43	6.55	0.39 **	0.37 **	0.27 **	0.35 **	−0.47 **	1

* *p* < 0.05 (two-tailed); ** *p* < 0.01 (two-tailed).

**Table 3:** Bootstrap test results of mediation paths.

Path	β	95% CI
Total effect between family support and mental health	0.118	−0.064, 0.353
Direct effect between family support and mental health	0.045	−0.132, 0.298
Indirect effect between family support and mental health	0.073	−0.009, 0.233
Total effect between teacher support and mental health	0.128	−0.168, 0.368
Direct effect between teacher support and mental health	0.038	−0.266, 0.273
Indirect effect between teacher support and mental health	0.090	0.009, 0.220
Total effect between peer support and mental health	0.335	0.082, 0.615
Direct effect between peer support and mental health	0.258	0.002, 0.534
Indirect effect between peer support and mental health	0.077	−0.008, 0.189

## Data Availability

The data presented in this study are available on request from the corresponding author. The data are not publicly available due to privacy.

## References

[1] Ataiants J., Cohen C., Riley A.H., Lieberman J.T., Reidy M., Chilton M. (2018). Unaccompanied children at the United States border, a human rights crisis that can be addressed with policy change. J. Immigr. Minor Health.

[2] Zijlstra A.E., Menninga M.C., Van Os E.C.C., Rip J.A., Knorth E.J., Kalverboer M.E. (2019). ‘There is no mother to take care of you’. Views of unaccompanied children on healthcare, their mental health and rearing environment. Resid. Treat. Child. Youth.

[3] Ministry of Civil Affairs (2019). Opinions on Further Strengthening the Protection of de Facto Unaccompanied Children.

[4] Cao H. (2008). Analysis of social support networks for “unaccompanied children” in rural areas: Based on a survey in Huangpi and Anlu, Hubei province. Youth Explor..

[5] Guo J., Liu T. (2014). Analysis on the social assistance trouble and its causes for the abandoned children. Theory Horiz..

[6] Han L., Yuan J., Long Y. (2021). Will moss bloom like peonies? The relationship between negative life events and mental health of left-behind children. Psychol. Dev. Educ..

[7] He B., Fan J., Liu N., Li H., Wang Y., Williams J., Wong K. (2012). Depression risk of ‘left-behind children’ in rural China. Psychiatry Res..

[8] Wang L., Feng Z., Yang G., Yang Y., Dai Q., Hu C., Liu K., Guang Y., Zhang R., Xia F. (2015). The epidemiological characteristics of depressive symptoms in the left-behind children and adolescents of Chongqing in China. J. Affect. Disord..

[9] Bronstein I., Montgomery P., Ott E. (2013). Emotional and behavioural problems amongst Afghan unaccompanied asylum-seeking children: Results from a large-scale cross-sectional study. Eur. Child Adolesc. Psychiatry.

[10] Herz M., Lalander P. (2017). Being alone or becoming lonely? The complexity of portraying ‘unaccompanied children’ as being alone in Sweden. J. Youth Stud..

[11] Whetten K., Ostermann J., Pence B.W., Whetten R.A., Messer L.C., Ariely S., O’Donnell K., Wasonga A.I., Vann V., Itemba D. (2014). Three-year change in the wellbeing of orphaned and separated children in institutional and family-based care settings in five low-and middle-income countries. PLoS ONE.

[12] Whetten K., Ostermann J., Whetten R., O’Donnell K., Thielman N. (2011). More than the loss of a parent: Potentially traumatic events among orphaned and abandoned children. J. Trauma. Stress.

[13] Whetten K., Ostermann J., Whetten R.A., Pence B.W., O’Donnell K., Messer L.C., Thielman N.M. (2009). A comparison of the wellbeing of orphans and abandoned children ages 6-12 in institutional and community-based care settings in 5 less wealthy nations. PLoS ONE.

[14] Bai Z. (2013). Implementation of the Strategy of Group Work Involved Who Have Lost Children’s Bad Behavior Correction—An Example to Explore the “Warm Group”. Master’s Thesis.

[15] Li L. (2020). Perceived Loss of Unaccompanied Minor from the Perspective of Resilience. Master’s Thesis.

[16] Peng Y. (2020). The Adversity and Resilience of Support-Less Children. Master’s Thesis.

[17] Henderson S., Byrne D.G., Duncan-Jones P. (1981). Neurosis and the Social Environment.

[18] Sarason I.G., Levine H.M., Basham R.B., Sarason B.R. (1983). Assessing social support: The Social Support Questionnaire. J. Personal. Soc. Psychol..

[19] Fan X., Lu M. (2020). Testing the effect of perceived social support on left-behind children’s mental well-being in mainland China: The mediation role of resilience. Child. Youth Serv. Rev..

[20] Nyoni T., Nabunya P., Ssewamala F.M. (2019). Perceived social support and psychological wellbeing of children orphaned by HIV/AIDS in Southwestern Uganda. Vulnerable Child. Youth Stud..

[21] Li X. (2021). A study on guarantee mechanisms for unaccompanied children. J. Tast. Class..

[22] McMahon G., Creaven A.M., Gallagher S. (2020). Stressful life events and adolescent well-being: The role of parent and peer relationships. Stress Health.

[23] Wang J., Li A., Nie J. (2020). The parallel mediating role of self-stigma and resilience in the relationship between attachment and mental health of junior high school orphan students. Psychol. Dev. Educ..

[24] Zhao L. (2019). Research on Social Support and School Adjustment of Left-Behind Children in Rural China. Ph.D. Thesis.

[25] Zhao J. (2021). An investigation of the impact of boarding schools on the education of rural left-behind children: A case study of boarding junior high schools. Hebei Educ. Compr. Ed..

[26] Bronfenbrenner U., Morris P.A., Damon W., Lerner R.M. (2006). Handbook of child psychology. The Bioecological Model of Human Development.

[27] Erikson E.H. (1964). Childhood and Society.

[28] Ven P. (2020). The journey of sensemaking and identity construction in the aftermath of trauma: Peer support as a vehicle for coconstruction. J. Community Psychol..

[29] Harris J.R. (1995). Where is the child’s environment? A group socialization theory of development. Psychol. Rev..

[30] Yeates K.O., Gerhardt C.A., Bigler E.D., Abildskov T., Dennis M., Rubin K.H., Stancin T., Taylor H.G., Vannatta K. (2013). Peer relationships of children with traumatic brain injury. J. Int. Neuropsychol. Soc..

[31] Zou H. (1998). The developmental function and influencing factors of peer relationships. Psychol. Dev. Educ..

[32] Llorca-Mestre A., Samper-García P., Malonda-Vidal E., Cortés-Tomás M.T. (2017). Parenting style and peer attachment as predictors of emotional instability in children. Soc. Behav. Personal. Int. J..

[33] Rubin K.H., Dwyer K.M., Kim A.H., Burgess K.B., Booth-Laforce C., Rose-Krasnor L. (2004). Attachment, friendship, and psychosocial functioning in early adolescence. J. Early Adolesc..

[34] Wang D., Yang X., Wang S., Chen C., Sun M., Zhang W., Pu W., Ouyang X., Liu Z. (2019). Peer attachment moderates the relationship between psychological resilience and psychotic-like experiences in left-behind children. Chin. J. Clin. Psychol..

[35] Zhao J., Liu X., Zhang W. (2013). Peer rejection, peer acceptance and psychological adjustment of left-behind children: The roles of parental cohesion and children’s cultural beliefs about adversity. Acta Psychol. Sin..

[36] Kumpfer K.L., Glantz M.D., Johnson J.L. (1999). Resilience and development: Positive life adaptations. Factors and Processes Contributing to Resilience: The Resilience Framework.

[37] Andrews F.M., Withey S.B. (1974). Developing measures of perceived life quality: Results from several national surveys. Soc. Indic. Res..

[38] Kovacs M. (1985). The children’s depression inventory (CDI). Psychopharmacol. Bull..

[39] Wang J., Zhang H., Hu H., Chen L., Zhang Z., Yu F., Li W., Wei S. (2010). Reliability and validity testing on the child depression inventory in Hefei. Mod. Prev. Med..

[40] Campbell-Sills L., Stein M.B. (2007). Psychometric analysis and refinement of the Connor-davidson Resilience Scale (CD-RISC): Validation of a 10-item measure of resilience. J. Trauma. Stress.

[41] Wang L., Shi Z., Zhang Y., Zhang Z. (2010). Psychometric properties of the 10-item Connor-Davidson Resilience Scale in Chinese earthquake victims. Psychiatry Clin. Neurosci..

[42] Weston R., Gore P.A. (2006). A brief guide to structural equation modeling. Couns. Psychol..

[43] Hu L., Bentler P.M. (1999). Cutoff criteria for fit indexes in covariance structure analysis: Conventional criteria versus new alternatives. Struct. Equ. Model. A Multidiscip. J..

[44] Mallinckrodt B., Abraham W.T., Wei M., Russell D.W. (2006). Advances in testing the statistical significance of mediation effects. J. Couns. Psychol..

[45] Ai H., Hu J. (2016). Psychological resilience moderates the impact of social support on loneliness of “left-behind” children. J. Health Psychol..

[46] Mitra R., Hodes M. (2019). Prevention of psychological distress and promotion of resilience amongst unaccompanied refugee minors in resettlement countries. Child Care Health Dev..

[47] Kohut H., Goldberg A., Stepansky P.E. (1984). How Does Analysis Cure?.

